# Beneficial effects of voluntary wheel running on activity rhythms, metabolic state, and affect in a diurnal model of circadian disruption

**DOI:** 10.1038/s41598-022-06408-z

**Published:** 2022-02-14

**Authors:** Carmel Bilu, Haim Einat, Paul Zimmet, Vicktoria Vishnevskia-Dai, William J. Schwartz, Noga Kronfeld-Schor

**Affiliations:** 1 School of Zoology, Tel Aviv University, 69978 Tel Aviv, Ramat Aviv, Israel; 2 School of Behavioral Sciences, Tel Aviv-Yaffo Academic College, Tel Aviv, Israel; 3 Department of Medicine, Monash University, Melbourne, VIC Australia; 4 Ocular Oncology and Autoimmune Service, The Goldschleger Eye Institute, The Chaim Sheba Medical Center, Tel-Hashomer, Sackler Faculty of Medicine, Tel Aviv University, Tel Aviv, Israel; 5 Department of Neurology, Dell Medical School, The University of Texas at Austin, Austin, TX USA; 6 Department of Integrative Biology, College of Natural Sciences, The University of Texas at Austin, Austin, TX USA

**Keywords:** Type 2 diabetes, Circadian rhythms

## Abstract

Emerging evidence suggests that disruption of circadian rhythmicity contributes to development of comorbid depression, cardiovascular diseases (CVD), and type 2 diabetes mellitus (T2DM). Physical exercise synchronizes the circadian system and has ameliorating effects on the depression- and anxiety-like phenotype induced by circadian disruption in mice and sand rats. We explored the beneficial effects of voluntary wheel running on daily rhythms, and the development of depression, T2DM, and CVD in a diurnal animal model, the fat sand rat (*Psammomys obesus*). Voluntary exercise strengthened general activity rhythms, improved memory and lowered anxiety- and depressive-like behaviors, enhanced oral glucose tolerance, and decreased plasma insulin levels and liver weight. Animals with access to a running wheel had larger heart weight and heart/body weight ratio, and thicker left ventricular wall. Our results demonstrate that exercising ameliorates pathological-like daily rhythms in activity and blood glucose levels, glucose tolerance and depressive- and anxiety-like behaviors in the sand rat model, supporting the important role of physical activity in modulating the “circadian syndrome” and circadian rhythm-related diseases. We suggest that the utilization of a diurnal rodent animal model may offer an effective way to further explore metabolic, cardiovascular, and affective-like behavioral changes related to chronodisruption and their underlying mechanisms.

## Introduction

The comorbidity between depression, cardiovascular diseases (CVD), and type 2 diabetes mellitus (T2DM) has been repeatedly described in the literature^1–4^. Emerging evidence suggests that disruption of circadian rhythmicity may contribute to the development of these comorbidities, and we have proposed the term "circadian syndrome" for this combined pathological condition^5,6^.

Circadian rhythms are the manifestation of an internal timekeeping system (the “circadian clock*”*) that is re-set (entrained) through daily environmental timing cues, especially light but also feeding and physical activity, allowing living things to anticipate periodic daily events, to orchestrate internal temporal programs of behavioral and physiological functions, and to flexibly set the order and scheduling of such functions to optimize fitness in the real world. In mammals, a “central” or “master” clock in the hypothalamus of the brain (suprachiasmatic nucleus [SCN]) synchronizes “peripheral” clocks found in cells, tissues, and organs throughout the brain and body to regulate metabolism and temporal physiology^7,8^. This network of clocks is capable of adaptively re-aligning its oscillatory components under changing conditions, including to signals such as, e.g., temperature, blood glucose and oxygen levels, and glucocorticoids. Importantly, there is increasing recognition that pathological misalignment of network components—between the environment, behavior, SCN, and peripheral clocks (e.g., in shiftwork and jet lag) —has critical implications not only for an individual’s health and performance but also for the pathophysiology of aging and disease^9–12^. Such circadian disruption in animal models, as well as in humans, may lead to the “circadian syndrome,” with depressive-like behavior^13^, CVD^9^, and T2DM^13^.

We have been investigating this syndrome in a unique animal model, the fat sand rat (*Psammomys obesus*). Sand rats are diurnally active in nature^14^, but when kept indoors under standard laboratory conditions they demonstrate an unstable, nocturnal phase preference, with low amplitude, and in some cases, no rhythm at all^5,15,16^. Interestingly, similar findings have been reported for almost all diurnal species examined to date^17–22^; conversely, such circadian disruption under normal laboratory conditions has not been documented in nocturnal rodents^16,22^. The laboratory-associated disruption of circadian rhythmicity in diurnal rodents is even more pronounced when animals are kept under a short photoperiod regimen (5 h light:19 h dark)^15,23^. Within approximately 8 weeks of short photoperiod acclimation with standard rodent diet, sand rats develop the "circadian syndrome," manifesting as glucose intolerance, elevated plasma insulin levels, cataracts, CVD, and depressive- and anxiety-like behaviors^5,6^. Strong entrainment of the circadian system by keeping sand rats outdoors, where a wealth of biotic and abiotic variables cycle, or by bright light treatment, prevents the development of these disorders^15,23,24^.

Notably, the depression- and anxiety-like phenotype induced by circadian disruption in mice and in sand rats is ameliorated upon provision of a running wheel^25,26^, and wheel running is known to have synchronizing effects on the entrainment of circadian systems^25,27,28^. Also, faster recovery of internal synchrony occurs following light/dark shift, and increased amplitude of SCN firing rates is seen compared to aged mice housed without a running wheel^29^. The effects of wheel running are likely complex^29^, but at least in part, effects on the circadian system may be mediated through the effects of exercise on skeletal muscles^10^. Skeletal muscle and bone have roles extending beyond regulation of locomotion and postural support, including the control of nutritional homeostasis, such as maintaining glucose and calcium levels. Feeding and exercise stimulate skeletal muscle tissues and change their functions, including the maintenance of tissue mass and metabolism^30^. It is thought that through these interactions with skeletal muscles, exercise may regulate circadian factors that influence mental, metabolic, and cardiovascular health. For instance, deregulated circadian rhythms in skeletal muscles are associated with reduced glucose tolerance, as well as increased rates of diabetes and CVD^31,32^.

To further explore these physiological effects on circadian rhythmicity and health, we have tested the influence of voluntary wheel running on the development of the full “circadian syndrome” in the fat sand rat model. Our results show that this rhythmic intervention has significant salutary effects on the metabolic and affective complications of the syndrome.

## Results

### General locomotor activity rhythm

**Figure 1:**
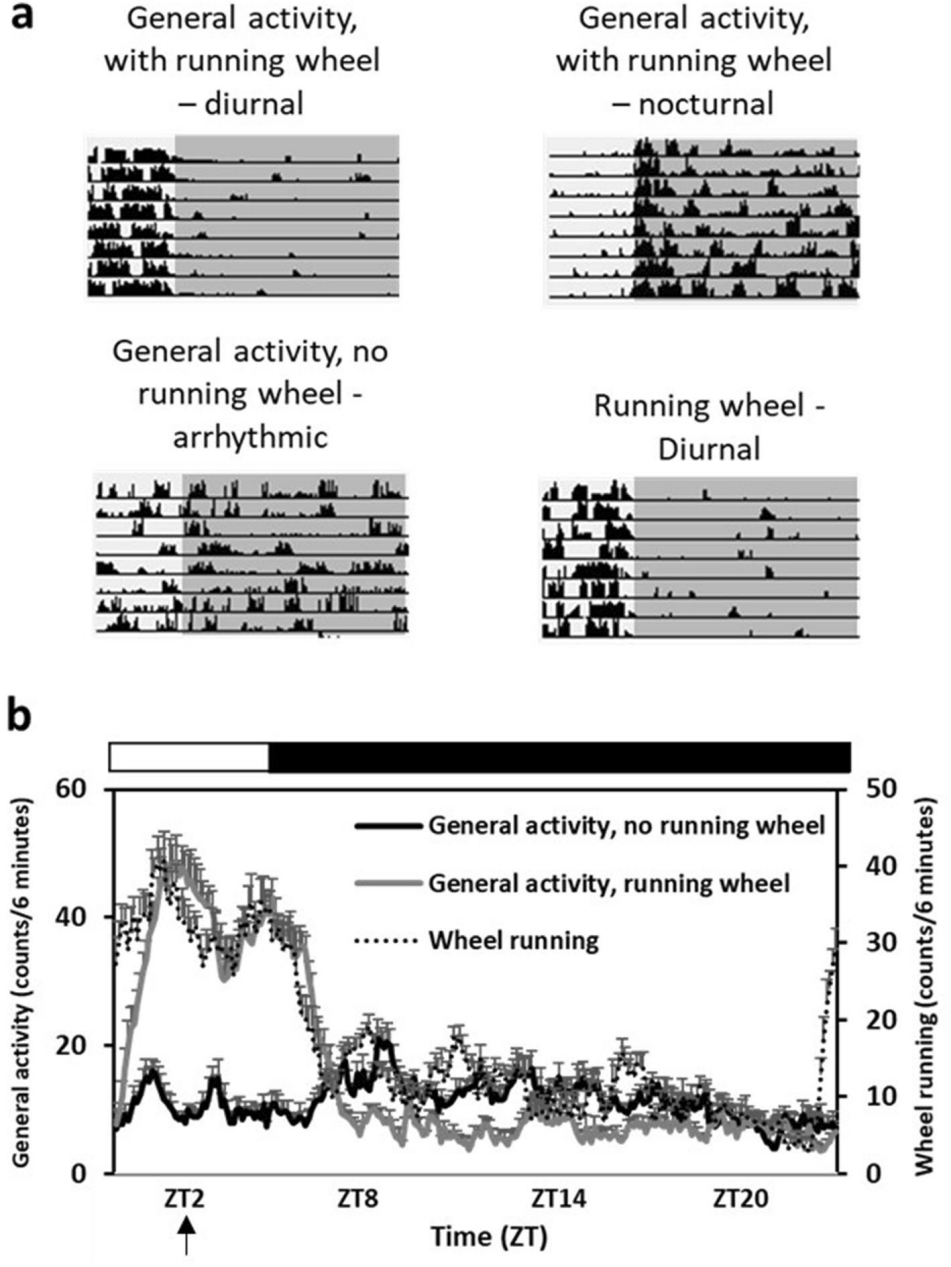
General locomotor activity patterns of the different treatment groups during the experiment. (**a**) Representative actograms of sand rats from each experimental group. Each row represents one day, depicted one below the other. Dark background represents dark hours. (**b**) Average daily activity rhythm of sand rats with and without a running wheel, and average running activity in the wheel, during weeks 7–8. N = 10–12 per group, Mean ± SEM. The black bar above the figure represents dark hours, the white bar—light hours. Bottom arrow represents the time when oral glucose tolerance and plasma insulin were measured, and when behavioral tests were performed.

Sand rats kept without access to running wheels (No wheels) were all arrhythmic (no significant rhythm detected in a χ2 test, see methods) [12/12 arrhythmic], whereas sand rats kept with running heels were either diurnal (5/10, more than 50% of their activity occurred during the light phase), nocturnal (2/10, more than 50% of their activity occurred during the dark phase), or arrhythmic (3/10) [χ^2^(1) = 4.91, p = 0.027] (Fig. 1).

### Oral glucose tolerance test

**Figure 2:**
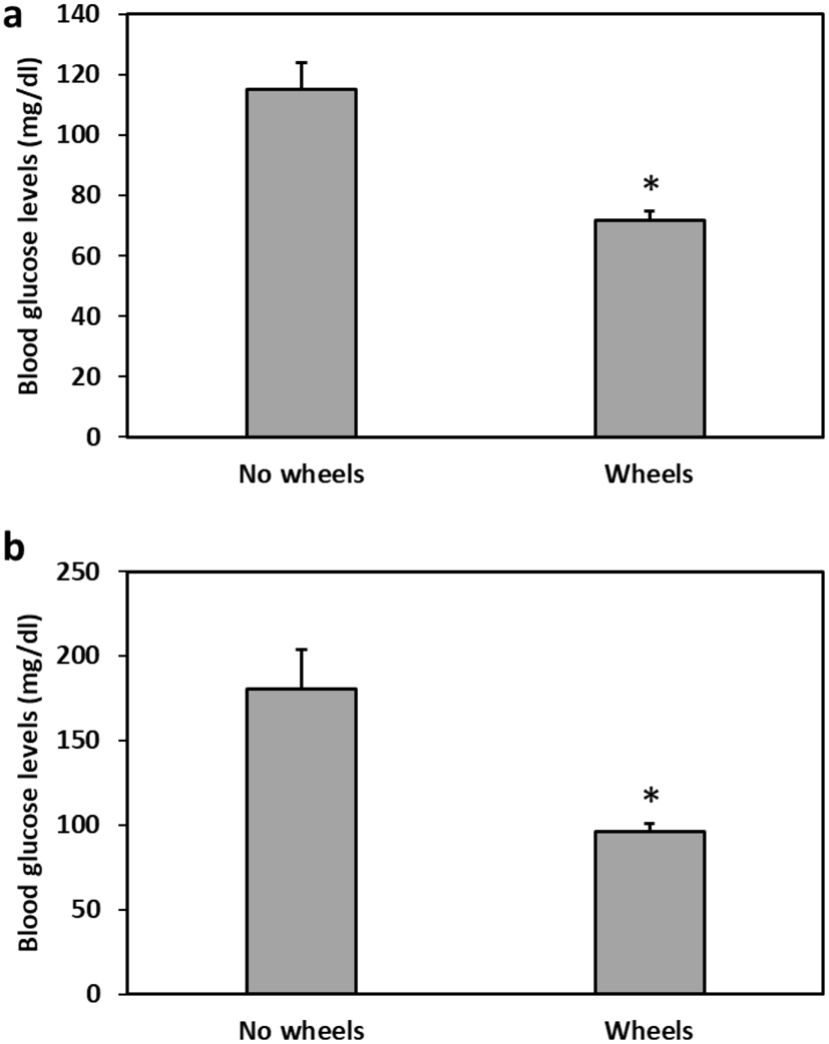
Effects of voluntary wheel running on baseline glucose levels and on glucose tolerance. The No wheels animals showed significantly higher blood glucose levels than the Wheels group both at baseline (**a**) and 120 min after oral glucose administration in the oral glucose tolerance test (**b**). *p < 0.005. N = 10–12 per group, Mean ± SEM.

The presence of a running wheel in the cage had a significant effect on baseline blood glucose levels and on glucose tolerance, with the No wheels group showing significantly higher blood glucose levels than the Wheels group both at baseline (T-test, t = − 4.2, p = 0.0004) and 120 min after oral glucose administration in the oral glucose tolerance test (T-test, t = − 3.31, p = 0.004) (Fig. 2).

### 24-h blood glucose rhythm

**Figure 3:**
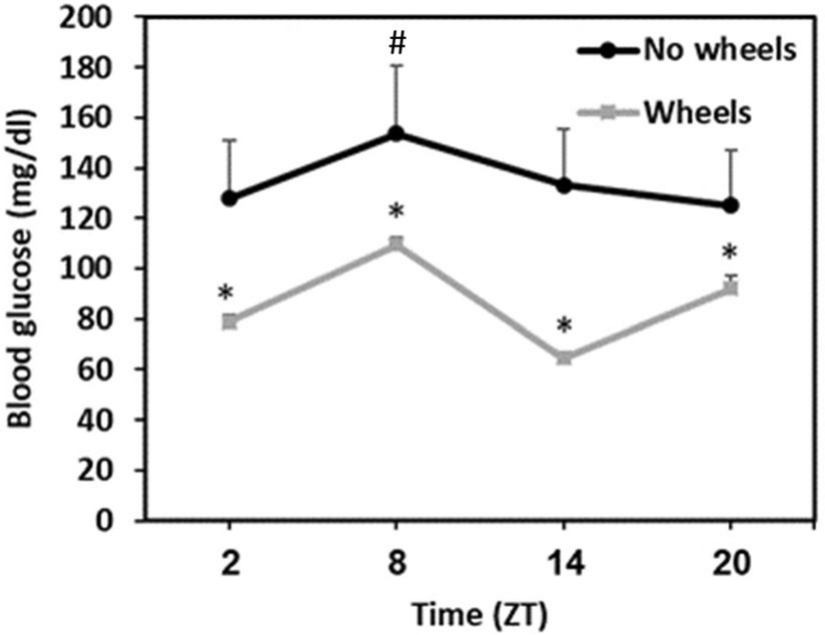
24-h blood glucose rhythm. Wheels animals showed a more pronounced daily rhythm in blood glucose levels. *Signifies a significant difference from all other ZTs (p < 0.001). # signifies a significant difference from ZT2 and ZT20. N = 10–12 per group, Mean ± SEM.

No wheels sand rats showed some daily glucose rhythm with levels at ZT8 different than levels at ZT2 and ZT20 [Repeated measures ANOVA, F(3, 33) = 4.53, p = 0.01; post-hoc, p = 0.009]. Wheels sand rats demonstrated a more pronounced rhythm, with significant differences between all ZT points [Repeated measures ANOVA, F(3, 27) = 47.8, p =  < 0.0001; post-hoc, each ZT is different from all other ZTs, p = 0.0001] (Fig. 3).

### Plasma insulin

**Figure 4:**
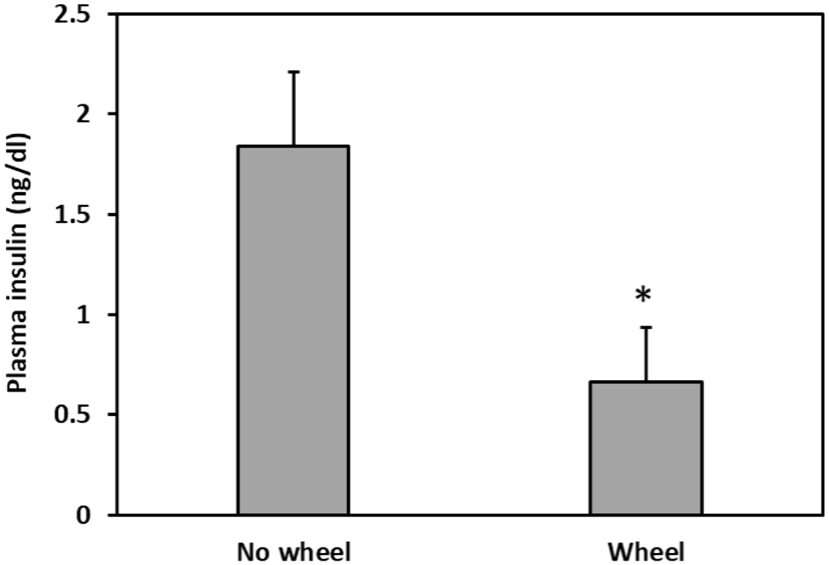
Plasma insulin levels. No wheels sand rats showed significantly higher plasma insulin levels than Wheels sand rats. *signifies p < 0.02. N = 10–12 per group, Mean ± SEM.

Wheel presence in the cage had a significant effect on plasma insulin levels, with No wheels sand rats showing significantly higher plasma insulin levels than Wheels animals (T-test, t = -2.53, p = 0.0199) (Fig. 4).

### Cataracts

The presence of a wheel in the cage had no significant effect on the development of cataracts [χ2(1) = 2.95, p = 0.086]. Nevertheless, 4/12 of the No wheels animals had mature cataracts versus none in the Wheels group (0/10).

### Body weight

There was no effect of wheel presence in the cage on body weight at the end of the experiment (week 11) (T-test, t = − 1.045, p = 0.31).

### Heart weight, heart/body weight, and left ventricle wall thickness

**Figure 5:**
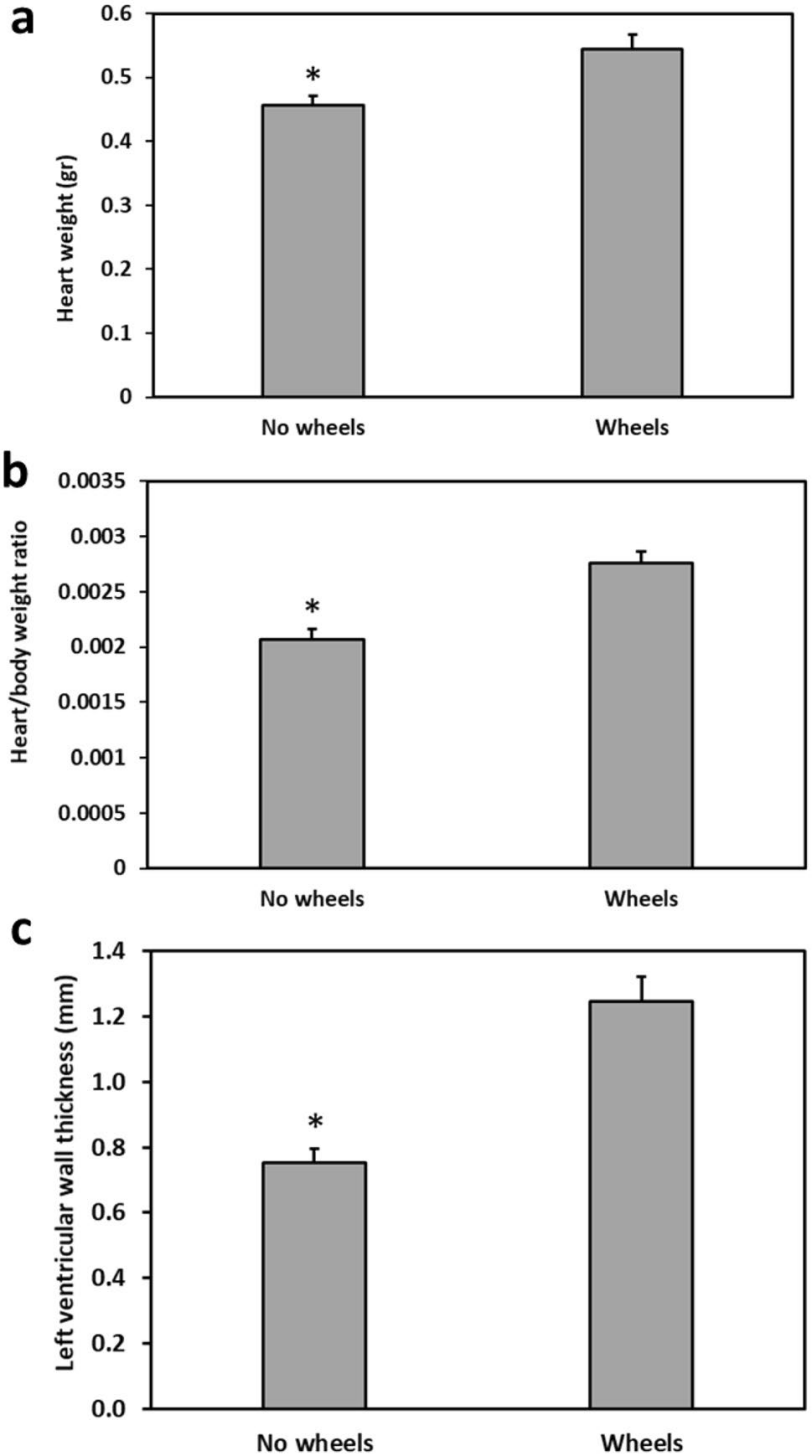
Effect of wheel presence in the cage on heart weight, heart/body weight ratio, and left ventricular wall thickness. Wheels animals show larger heart weight (**a**), larger heart/body weight ratio (**b**), and a thicker left ventricular wall (**c**) than No wheels animals. *Signifies p < 0.005. N = 10–12 per group, Mean ± SEM.

The heart weight and heart/body weight ratio were larger in Wheels animals compared to No wheels animals (T-test, Heart weight: t = 3.34, p = 0.0035, Fig. 5a; heart/body weight ratio: t = 4.823, p = 0.0001, Fig. 5b).

The left ventricular wall was thicker in Wheels animals than in No wheels animals (T-test, t = 5.25, p = 0.00005) (Fig. 5c).

### Liver weight

**Figure 6:**
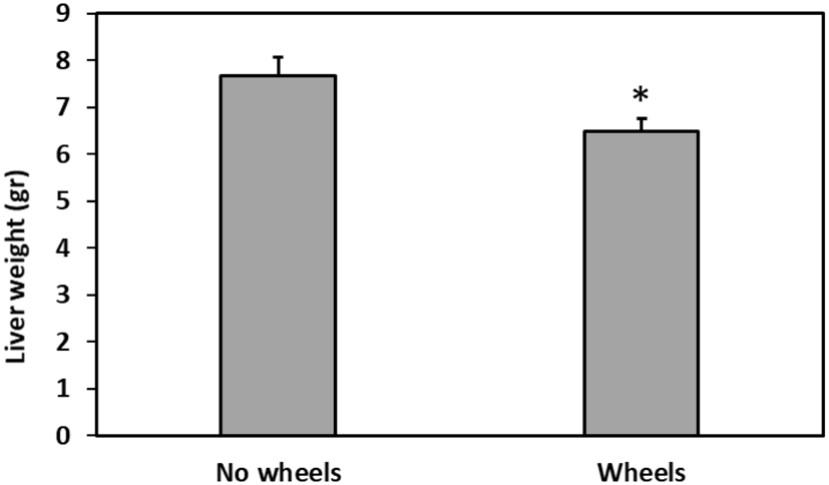
Effect of wheel presence in the cage on liver weight. No wheels animals show a higher liver weight than Wheels animals. *Signifies p < 0.03. N = 10–12 per group, Mean ± SEM.

Liver weight was greater in No wheels animals compared to Wheels animals (T-test, t = − 2.45, p = 0.0241) (Fig. 6). No difference between the groups was found in liver/body weight ratio (T-test, t = − 0.728, p = 0.4755).

### Behavioral tests

**Figure 7:**
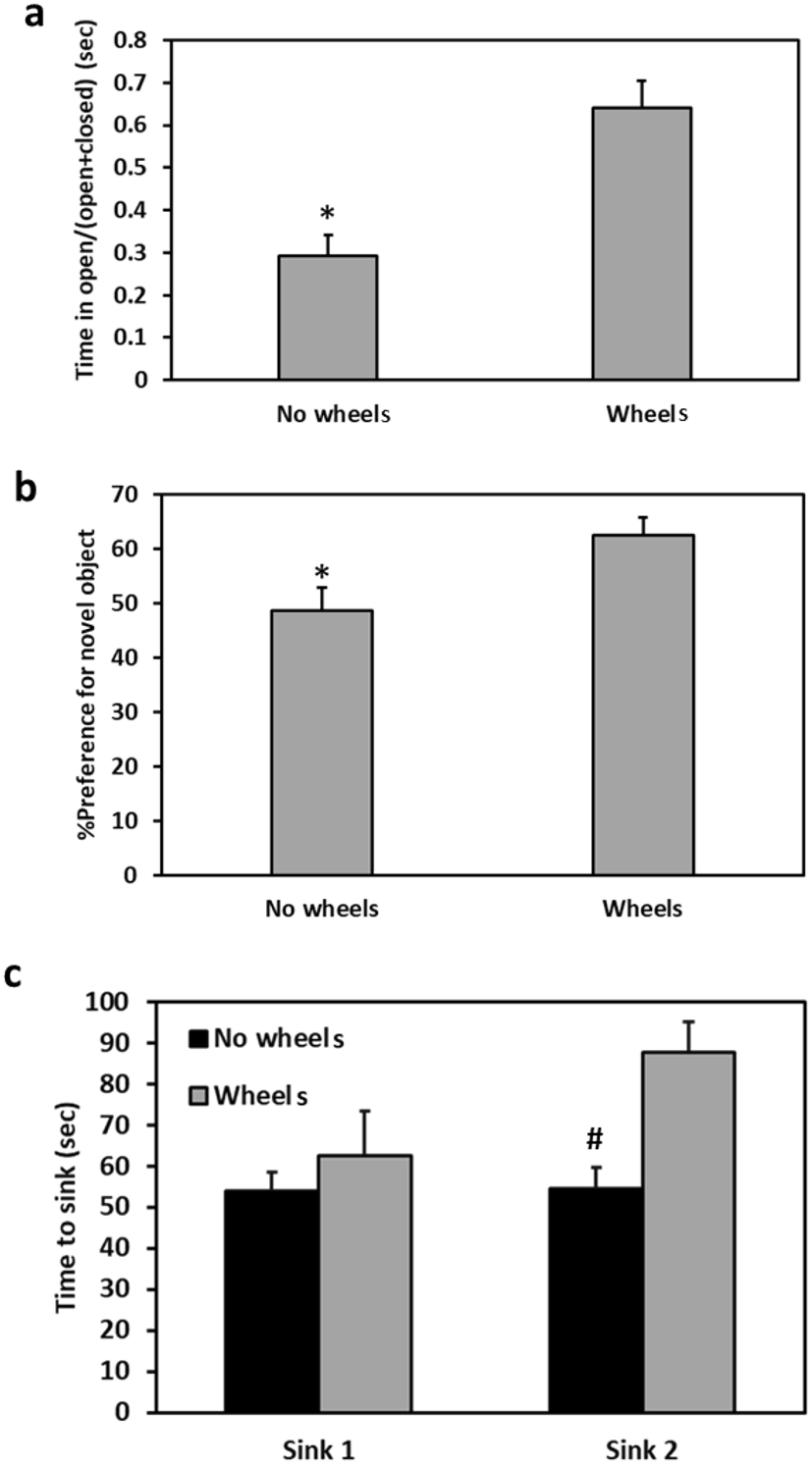
Effect of wheel presence in the cage on behavior. No wheels animals show a significantly shorter time spent in the open arms of the maze in the EPM test (**a**), lower preferential exploration of the novel object in the NORT (**b**), and shorter time to the first and second sink in the FST (**c**), compared with Wheels animals. *Signifies p < 0.02. # signifies p < 0.005 between the No wheels group and the Wheels group in the second sink (Sink 2). N = 10–12 per group, Mean ± SEM.

Measuring anxiety-like behavior in the EPM, we found that animals maintained without running wheels spent less time in the open arms of the EPM than animals maintained with running wheels, as demonstrated by lower open/total time ratio measure (T-test, t = 6.123, p = 0.00001) (Fig. 7a). There was no difference between the groups in the number of entries to each arm of the maze (T-test, t = 0.43, p = 0.6718]. Wheels animals showed higher preferential exploration of the novel object during the NORT than No wheels animals (T-test, t = 2.536, p = 0.02) (Fig. 7b). For the Wheels group the interaction time with the familiar object was 107.7 ± 8.5 s and with the novel object 181.0 ± 11.3 s. For the No wheels group: familiar object—119.6 ± 16.7 s and novel object—115.3 ± 13.9 s (T-test for interaction with familiar object, t = 3.558, p = 0.002).

The Wheels group showed a longer time to sink in the FST with near significant effect in the first sink [ANOVA, wheels effect F(1, 20) = 4.31, p = 0.051] and a significant effect in the second sink [ANOVA, wheels effect F(1, 20) = 4.81, p = 0.04] (Fig. 7c).

### Influence of general activity level

To examine the possibility that outcomes were influenced by the level of general activity rather than wheel effects on circadian rhythmicity, we computed the mean total activity per 24 h for animals maintained with wheels, and analyzed possible correlations between this measure and the behavioral and physiological measures described above. For the behavioral measures there were no significant correlations between activity and EPM open/closed ratio (r = 0.21, p = 0.56), FST sink 2 (r = 0.18, p = 0.61), or NORT discrimination ratio (r = 0.58, p = 0.08). For the physiological measures, there was only one significant correlation, between activity and glucose levels (r = 0.82, p = 0.004) but no significant correlations with insulin levels (r = 0.03, p = 0.94), heart/body weight ratio (r = 0.42, p = 0.23) or liver weight (r = 0.52, p = 0.13).

## Discussion

Our results show that voluntary wheel running for 11 weeks inhibits the development of the “circadian syndrome” in fat sand rats housed indoors in standard laboratory conditions. Daily rhythms of general locomotor activity and blood glucose are strengthened, blood glucose and plasma insulin levels are lowered, oral glucose tolerance is enhanced, and tests of depressive- and anxiety-like behaviors and memory are improved.

We found that sand rats kept without access to a running wheel were all behaviorally arrhythmic, whereas most sand rats kept with a running wheel showed a significant daily locomotor activity rhythm (7/10) and were either diurnal (5/10) or nocturnal (2/10), while only 3/10 were arrhythmic (Fig. 1). These results are consistent with those of an earlier study which showed that 3 weeks of voluntary wheel running had an enhancing effect on the activity rhythms of sand rats maintained under short photoperiod. Krivisky et al. (2015)^25^ found that activity patterns of sand rats were more robust and less fragmented when they had free access to a running wheel, and the effect was more pronounced in animals acclimated to a short photoperiod. Of note, physical exercise elevates the arterial CO_2_ tension (P_aCO2_)^33^, and Adamovich et al. (2019)^34^ found that changes in carbon dioxide levels altered the expression of several core circadian clock genes and shifted circadian phase in cultured cells. We speculate that one mechanism by which voluntary exercise could influence the circadian system might be through the modulation of P_aCO2_.

Voluntary wheel running had a significant effect on baseline blood glucose levels and on glucose tolerance, with the No wheels group showing significantly higher blood glucose levels than the Wheels group, both at baseline and 120 min after oral glucose administration in the oral glucose tolerance test (Fig. 2). Furthermore, the blood glucose rhythm demonstrated by the Wheels group was much more pronounced compared with the No wheels group (Fig. 3). Accordingly, plasma insulin levels were lower in the Wheels group compared to the No wheels group (Fig. 4). These results are in line with previous findings showing that treatments which enhance and strengthen the circadian system in sand rats prevent the development of T2DM that otherwise occurs under standard laboratory conditions. For example, Bilu et al. (2019)^20^ showed that when kept outdoors in laboratory cages, sand rats were diurnal, demonstrating robust daily rhythms in blood glucose levels, and did not develop T2DM. This contrasted with sand rats kept indoors under similar conditions, which became nocturnal or arrhythmic, had constantly higher blood glucose levels with no significant daily rhythmicity, and developed glucose intolerance^15^. Along this line, Bilu et al. (2020)^24^ found that sand rats subjected to morning bright light treatment, which enhances circadian rhythmicity^23,24^, showed robust daily rhythms in clock gene expression and behavior, and were normoglycemic, with higher glucose tolerance than the control group.

Blood glucose homeostasis is manifested by daily rhythmicity in glucose tolerance and in blood glucose levels^35^. At the beginning of the activity/feeding phase, when blood glucose is mainly of dietary origin^35^, glucose tolerance and blood glucose levels are at their highest^36^. During the resting/fasting period, glucose is recruited from endogenous gluconeogenesis in the liver, and glucose tolerance and blood glucose levels are lower^36^. Daily rhythmicity in blood glucose levels is regulated directly by the SCN and by peripheral clocks in the liver, pancreas, muscle, and white adipose tissue^35^. When the SCN is lesioned in rats, the daily fluctuation in glucose uptake and insulin sensitivity disappears^36^. Furthermore, mutations of clock genes cause disruption of glucose homeostasis, with severely disturbed daily blood glucose rhythms, hyperglycemia, and hypoinsulinemia^36–38^. However, rhythms in glucose tolerance and in blood glucose levels also emanate from behavioral rhythms in exercise and feeding^39,40^. Skeletal muscle is the major tissue responsible for insulin-mediated glucose utilization. The plasma membrane GLUT4 content is correlated with glucose transport activity in both animal models and human skeletal muscle^41^. Regular exercise results in elevated insulin- and contraction-stimulated glucose transport capacity by augmenting skeletal muscle GLUT4 levels^42^, and GLUT4 activity demonstrates circadian rhythmicity with higher expression levels and translocation during the activity phase than the rest phase^43^. In addition, feeding behavior modulates daily rhythms of blood glucose and glucagon concentrations^40^. The classic function of glucagon is to increase hepatic glucose output when glucose concentrations decline during fasting^40^. Ad libitum–fed rats show peaks in glucagon concentrations during the resting/fasting phase, shortly before the onset of the active/feeding phase^40^. In contrast, rats that start fasting at the onset of their activity phase have decreased plasma glucagon concentrations at the end of the resting period, instead of the expected increase^40^. Thus, further experiments will be required to determine the relative contributions of the SCN, peripheral clocks, and behavioral rhythms of exercise and feeding in accounting for the differences between the Wheels and the No wheels sand rats in glucose tolerance and blood glucose rhythmicity.

In contrast to our previous study^20^ reporting the development of mature cataracts in animals kept under short photoperiod conditions, we did not find a significant effect of wheel running on the development of mature cataracts (although 4/12 of the No wheels animals had mature cataracts, versus none in the Wheels group). This difference might be explained by the different length of the two experiments: Bilu et al. (2019)^15^ kept the sand rats under short photoperiod conditions for 20 weeks, whereas the current experiment lasted only 11 weeks, a time which might not be sufficient for the development of mature cataracts in these animals.

Another contrast with previous studies was the difference in heart weight, heart/body weight, and left ventricular wall thickness between the groups. In earlier studies we found that short photoperiod acclimated sand rats had a larger heart weight and heart/body weight ratio than neutral photoperiod acclimated sand rats^15^, and that animals with diabetes had a larger heart weight than non-diabetic sand rats^5^. We ascribed these results to the pathological cardiovascular effects of hyperglycemia, such as elevated aortic stiffness and reduced myocardial metabolism, which alters diastolic function in patients with diabetes mellitus. Furthermore, higher fasting blood glucose levels and glycated hemoglobin are associated with abnormal left ventricular relaxation in people with diabetes^44^. However, in the current study, the heart weight and heart/body weight ratio were larger, and the left ventricular wall was thicker, in the Wheels group and not in the diabetic No wheels group (Fig. 5). These results, which suggest left ventricular hypertrophy (LVH) in the Wheels group, could be explained by a physiological morphological adaptation to exercise ("athlete's heart")^45^, rather than the pathological LVH seen in the previous studies of diabetic sand rats^5,15,16^. In this case, since the Wheels group ran for about 5 h a day on their wheels, it is possible that the LVH we found is similar to that found in athletes participating in sporting disciplines involving intensive isometric exercise, like long-distance running^45^. These athletes may exhibit substantial physiological increases in average ventricular parietal mass, and in left ventricular (LV) parietal thickness, without impairment of contractile strength or LV performance^45^. However, in order to differentiate between a purely physiological, adaptive process vs. a pathological hypertrophic cardiomyopathy, further experiments will be needed to examine aspects of the sand rat heart's anatomical and functional characteristics, such as LV cavity size, LV diastolic function, and the absence of T-wave inversion on electrocardiography.

The higher liver weight in the No wheels group compared to the Wheels group (Fig. 6) is consistent with earlier findings of an increased liver size and abnormalities of liver enzymes in patients with diabetes mellitus^46^. During periods of hyperglycemia, glucose freely enters the hepatocytes, driving glycogen synthesis, and eventually causes the accumulation of excessive amounts of glycogen in the hepatocytes (glycogenosis), causing hepatomegaly and elevated aminotransferases. These abnormalities are readily reversible with sustained euglycemic control^47^. The other major cause of hepatomegaly in people with diabetes is steatosis. This is a function of the body habitus and state of insulin resistance, rather than glycemic control, and it may progress to fibrosis and cirrhosis^47^. In order to distinguish between these two causes of hepatomegaly, histological studies will be needed to assess for swollen hepatocytes containing excess glycogen in the cytoplasm, and often also in the nucleus. These are indicative of glycogenosis^48^. There was no significant difference in liver/body weight between the groups.

There has long been discussion regarding the ameliorating effects of exercise on cognitive functions and affect. However, exercise has only recently received the attention of the scientific community with the key interest in its effects on cognitive functions, affective disorders, spatial learning and memory, and as a non-drug method to maintain brain health^49^. Cross-sectional studies associate high self-reported levels of regular exercise with improved mental health and low depression^50^. Moreover, self-reported high levels of habitual physical activity correlate with fewer symptoms of both anxiety and depression and better mental health^51^. The neurobiological effects of exercise, acting as a mood elevating agent, appear to influence several neural mechanisms related to depression and anxiety^51^. There is evidence that physical activity causes physiological changes in monoamine levels^52^, upregulates neurotrophic factors^53^, alters the levels of the stress hormone cortisol^54^, and leads to adaptations in limbic structures implicated in depressive and anxiety disorders.

A relationship between circadian rhythmicity and affective disorders has been observed at the biochemical, molecular, and clinical levels. Manipulation of light exposure affects various neurotransmitter systems related to mood^55^, including adrenaline and serotonin^56^. Antidepressants and mood stabilizers affect systems related to circadian rhythmicity^57^, and modifications of genes related to circadian rhythms influence affective-like behaviours. However, the mechanisms underlying the relationship between circadian rhythmicity and affective disorders are still not yet fully understood. In the present experiment, we found that animals maintained without running wheels showed higher anxiety- and depressive-like behaviors compared to animals maintained with running wheels (Fig. 7a, b). These results replicate previous findings, showing that in sand rats maintained under short photoperiod conditions, under which they display increased anxiety- and depressive-like behaviors^58^, these disorders could be ameliorated by voluntary wheel running^25^. Similar effects of exercise on anxiety- and depressive-like behaviors were previously described in other animal models of depression, such as mice^59^, and rats^60^, as well as humans^61^. Whereas it is possible, at least in part, that the ameliorating effects of running wheels on the FST reported here could be the result of better physical fitness, this explanation seems unlikely to account for the entire profile of effects. We found no correlations within the Wheels group between the level of general activity and the measures obtained in the behavioral tests, arguing against the level of activity per se as an explanation for the behavioral results.

In addition to lower anxiety- and depressive-like behaviors, sand rats with access to running wheels showed higher preferential exploration of the novel object in the NORT, indicating better recognition memory (Fig. 7c). Notably, microarray studies have demonstrated that wheel running elevates hippocampal BDNF mRNA and protein levels, as well as the levels of its high-affinity receptor TrkB^62^. Upregulation of these molecules augments neurogenesis in the dentate gyrus of the hippocampus, a brain structure that is crucial for memory function, thus improving spatial learning and memory^63^. BDNF, its receptors, and epigenetic modulators are involved in the pathophysiology of affective disorders^64^, T2DM^65^, and circadian system function^66^. In an earlier study we found that acclimation to short photoperiod resulted in a diminished circadian rhythm of BDNF mRNA expression levels in the frontal cortex and SCN compared to neutral photoperiod. The sand rats that showed diminished BDNF circadian rhythms also demonstrated higher blood glucose and insulin levels, as well as significantly higher anxiety- and depressive-like behaviors compared to animals acclimated to neutral photoperiod. We speculated that BDNF may, at least in part, mediate the effects of circadian disruption on the development of the “circadian syndrome” in sand rats via the SIRT1-BDNF-Trkb pathway^67^.

In conclusion, our results demonstrate that voluntary wheel running ameliorates the metabolic and affective complications of the “circadian syndrome” in a diurnal animal model of circadian disruption, supporting an important role of rhythmic physical activity in modulating pathological features of the syndrome. The mechanisms mediating the beneficial effects of wheel running on components of the syndrome are likely complex and multifactorial, but we hypothesize that stabilization of disrupted circadian clock and rhythm function plays a key role. We suggest that the utilization of a diurnal rodent animal model offers an effective way to further analyze the metabolic, cardiovascular, and affective behavioral changes related to circadian disruption and their underlying mechanisms.

## Materials and methods

### Animals

24 male sand rats (*Psammomys obesus*, 6–7 months old, from our colony at Tel Aviv University Zoological Research Garden) were used as subjects. Animals were individually housed in standard plastic cages (30 cm ′ 40 cm ′ 40 cm), in temperature-controlled rooms (25 °C)^15^. After 3 weeks of acclimation, the sand rats were divided into two groups, based on weight and blood glucose levels to avoid a baseline bias, and kept under short photoperiod (5 h light:19 h dark, where we define ZT0 as lights-on): 12 sand rats were kept in cages with running wheels ("Wheels"), and 12 sand rats were kept in cages with no running wheels ("No wheels"). All animals were provided with *ad-lib* tap water and standard rodent food (product 19510; Koffolk, Petach-Tikva, Israel). Body weight was measured weekly during the experiment. In-cage general locomotor activity was monitored throughout the experiment, using IR motion detectors (Orev Ltd., Israel). Running activity in the wheels was recorded using inductive sensors (SI18-C, Aeco Sensors, Italy). Data were collected at 6-min intervals using designated software (ICPC, Netanya, Israel)^15^. Wheel running was detected as activity in the IR motion detectors. All experimental procedures followed the NIH guidelines for the care and use of laboratory animals and were approved by the Institutional Animal Care and Use Committee (IACUC) of Tel Aviv University (permit number L15055). The study is reported in accordance with ARRIVE guidelines.

### Procedure

Before the onset of the experiments, all animals were maintained on a low-energy diet (product 1078, Koffolk Ltd, Israel) and 12 h light:12 h dark cycle (lights on at 09:00 and off at 21:00), to prevent diabetes. All animals were weighed and tested for glucose tolerance before the start of the experiment. Animals were assigned to the experimental groups based on weight and blood glucose levels to avoid a baseline bias: 12 males in the Wheels group and 12 males in the No wheels group. Body weight was measured weekly during the experiment. On week 8 animals were evaluated in three standard behavioral tests of anxiety-like behavior (elevated plus-maze, EPM), memory (novel object recognition test, NORT) and depression-like behavior (forced swim test, FST). On week 9, around ZT 2, animals were weighed, blood was collected from the tail tips for glucose measurement (U-Right glucometer TD-4269, TaiDoc, New Taipei City, Taiwan), and oral glucose tolerance tests were performed. The plasma samples of 10 animals from each group were assayed for insulin. On week 10, all animals were tested for a 24-h blood glucose rhythm. Three days later, the animals’ eyes were examined for the presence of cataracts (a common complication of T2DM^68^). On week 11 the sand rats were euthanized around ZT 7 (during the dark phase), and left ventricle wall thickness and heart and liver weights were measured.

### Elevated plus-maze

Performed at ZT2, as described in Bilu et al. 2019^15^.

### Novel object recognition

The NORT, performed at ZT2, assesses recognition memory in animals. The experimental apparatus consisted of a white rectangular open field (75 cm × 55 cm × 40 cm). Habituation took place by exposing the animal to the experimental apparatus one time for 5 min in the absence of objects, on the day before training. During the training phase sand rats were placed in the experimental apparatus in the presence of two identical objects and allowed to explore for 15 min. After a retention interval of 24 h, animals were placed again in the apparatus, where this time one of the objects was replaced by a novel one. Sand rats were allowed to explore for 15 min. Preference for the novel object was expressed as the percent time spent exploring the novel object relative to the total time spent exploring both objects. The objects were a glass bottle and a rectangular plastic box, both with approximately the same height. The identity of the objects, which one was novel or familiar, as well as the spatial location (whether the novel object was placed on the left or right side during the test session) of each object was balanced between groups. A preference for either object was not observed in this study. Each group's ability to recognize the novel object was determined by dividing the mean time of the animal exploring the novel object by the mean of the total time exploring the novel and familiar objects during the test session (Tnovel/[Tnovel + Tfamiliar]). In both tasks, objects were rinsed with ethanol between trials and before the first trial. All testing and training sessions were videotaped and analyzed by an experimenter blind to the treatment of the animals. It was considered exploration of the objects when animals were facing and sniffing the objects within very close proximity and/or touching^69^.

### Forced swim test

Performed at ZT2, as described in Bilu et al. 2019^15^.

### Oral glucose tolerance test (GTT)

Performed in week 9, at ZT2, in animals fasted for 4 h, as described in Bilu et al. 2019^15^.

### Plasma insulin ELISA

Plasma insulin protein was assayed with an immunoassay ELISA kit (Rat Insulin Ultrasensitive, ALPCO, Salem, NH) on blood collected in week 9, at ZT2.

### 24-h blood glucose rhythm

On week 10, all animals were tested for a 24-h blood glucose rhythm by collecting blood from the tail tip at ZT2, ZT8, ZT14 and ZT20 and measuring blood glucose levels using a glucometer (U-Right glucometer TD-4269, TaiDoc, New Taipei City, Taiwan).

### Cataracts

Performed on week 10, as described in Bilu et al. 2019^15^.

### Heart weight, left ventricle wall thickness, and liver weight

On week 11, the sand rats were euthanized, the heart was collected as described in Bilu et al. 2019^15^. Subsequently, a section midway between the base and the apex, perpendicular to the longitudinal axis of the ventricle, was obtained to measure the average wall thickness of the free wall. Five equally spaced measurements of the left ventricular free wall were collected, and their values were averaged. Then, the liver was removed and rinsed in two washes of ice-cold saline. Major blood vessels and connective tissue were removed, the liver was blotted dry, and weighed.

### Statistical analysis

Statistical analysis was performed using Statistica 13.0 (Dell, Tulsa, OK). Data were analyzed using students’ t-tests or analysis of variance (ANOVA) as appropriate, and statistical significance was accepted at p < 0.05. Correlations were evaluated using Pearson’s Correlations. The actograms and the significance of the daily rhythm in general locomotor activity was calculated by χ2 test using CTools 7.0 software by van der Veen on data collected on week 10. Activity pattern was defined as diurnal if more than 50% of activity occurred during the light phase, and nocturnal if more than 50% of activity occurred during the dark phase. To identify the existence of daily rhythms in blood glucose levels we used one-way ANOVAs with ZT as main factor within each group to analyze differences between levels at different ZT points. Significant ANOVA results were followed by LSD post-hoc test. A rhythm was considered significant when there was a significant difference between at least two ZT time points^15^.

## Data Availability

The datasets generated during and/or analysed during the current study are available from the corresponding author on reasonable request.
